# Bull's-Eye Signs: Rapid Appearance of Diffuse Lung Cancer Metastases in the Stomach

**DOI:** 10.14309/crj.0000000000001366

**Published:** 2024-06-03

**Authors:** Daniella Mikhail, Ping He, Colton Smith, Anish Vinit Patel

**Affiliations:** 1 Department of Medicine, Rutgers Robert Wood Johnson Medical School, New Brunswick, NJ; 2 Division of Gastroenterology & Hepatology, Rutgers Robert Wood Johnson Medical School, New Brunswick, NJ; 3 Department of Pathology & Laboratory Medicine, Rutgers Robert Wood Johnson Medical School, New Brunswick, NJ

## CASE REPORT

**Figure 1.**
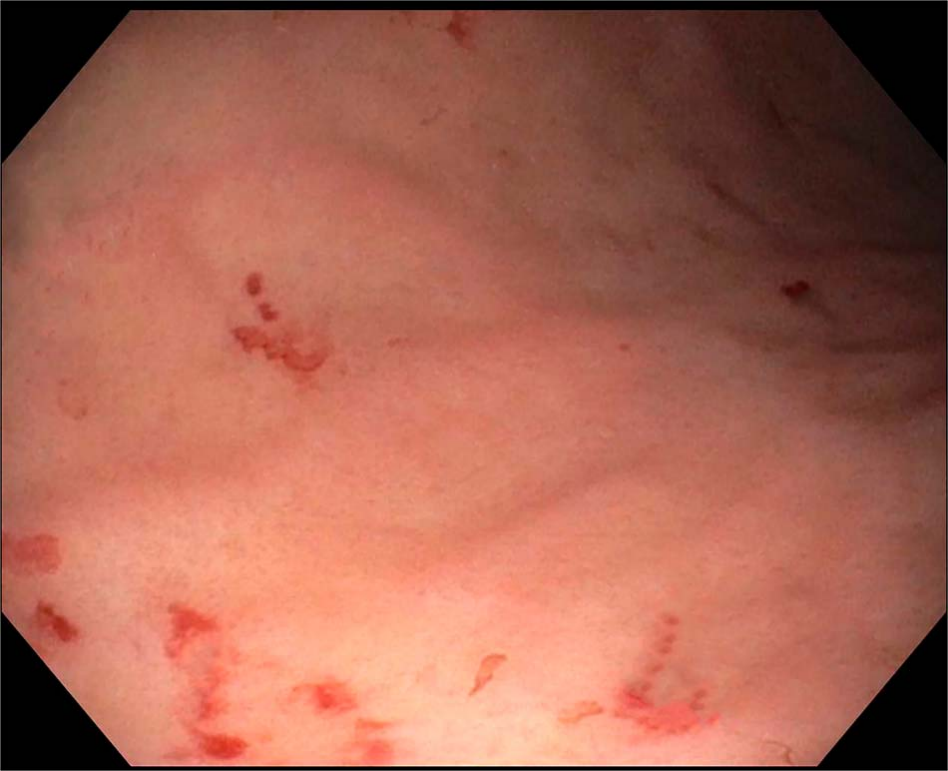
Previous esophagogastroduodenoscopy 2 weeks prior performed for evaluation of anemia with only findings of superficial gastritis, with biopsies showing chronic active inflammation.

**Figure 2.**
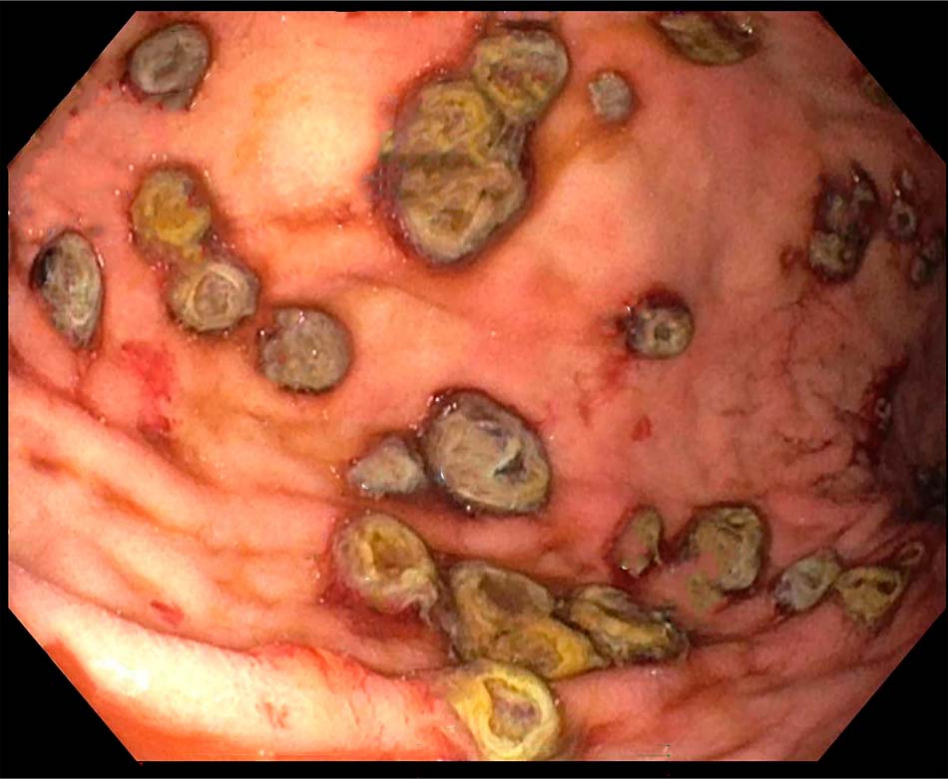
Esophagogastroduodenoscopy visualized numerous 5–10 mm-sized mucosal nodules scattered diffusely throughout the gastric body and antrum. The nodules demonstrated characteristic bull's-eye signs.

**Figure 3.**
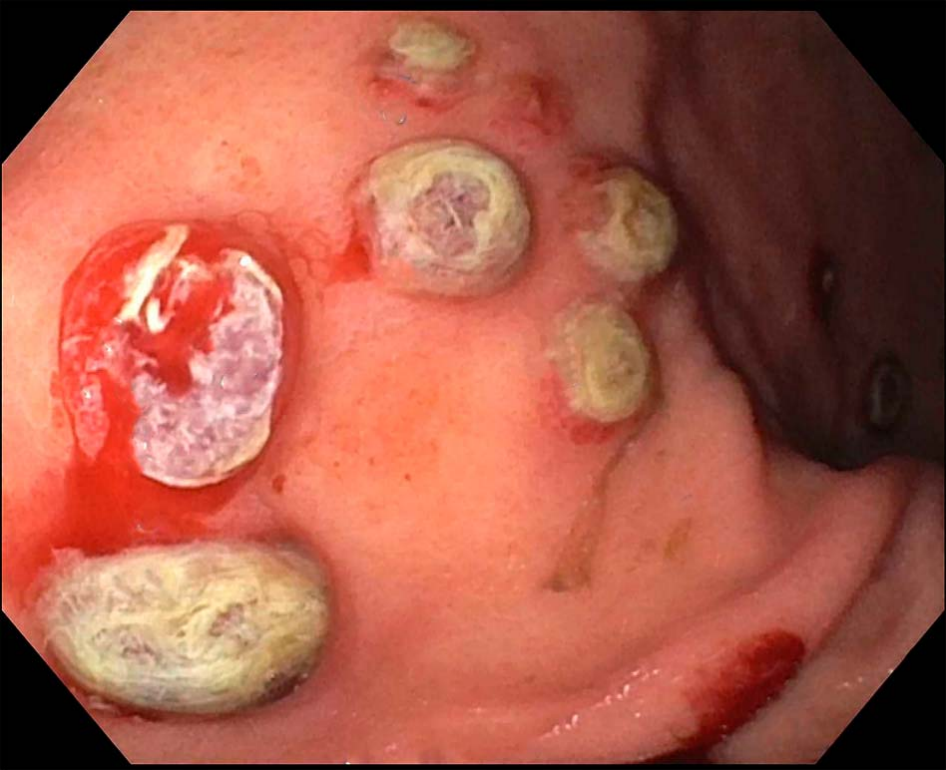
Mucosal nodules were biopsied for diagnosis.

**Figure 4.**
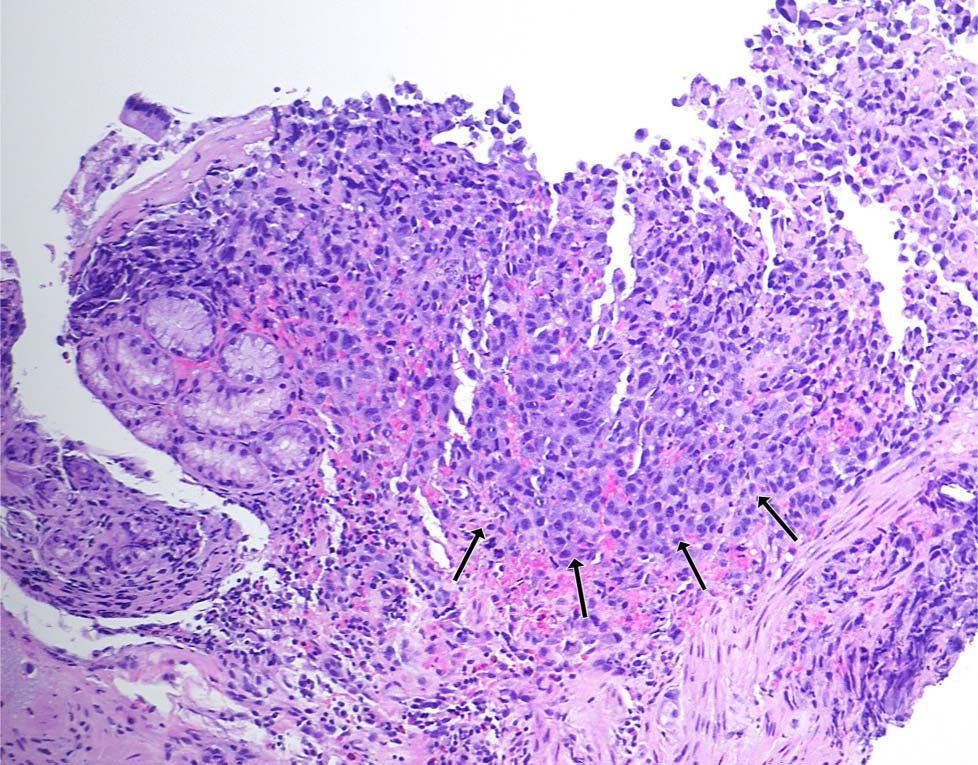
Biopsies showed infiltration of the gastric mucosa by a poorly differentiated adenocarcinoma (arrows) composed of large pleomorphic tumor cells with predominantly fine nuclear chromatin and prominent nucleoli (hematoxylin & eosin stain, 40×) with morphology identical to the patient's known lung adenocarcinoma—indicative of metastatic lesions.

A 50-year-old man was recently diagnosed with right upper lobe lung invasive poorly differentiated adenocarcinoma (95% programmed cell death ligand 1 positivity) with metastases to the adrenals and femur. He underwent an upper endoscopy for evaluation of normocytic anemia (hemoglobin 8.6 g/dL, iron 72 mcg/dL) with findings of superficial gastritis alone with biopsies showing chronic active inflammation (Figure 1). The patient then developed melena 2 weeks later with acute blood loss anemia. Hemoglobin declined to 5.9 g/dL with no iron deficiency. He did not receive chemotherapy or radiation therapy in the interim. A repeat upper endoscopy was performed and visualized numerous 5–10 mm-sized mucosal nodules scattered diffusely throughout the gastric body and antrum (Figures 2–3). The nodules demonstrated characteristic bull's-eye signs—central ulcerations covered by adherent fibrinous material. Biopsies were taken and returned as infiltration of the gastric mucosa by a poorly differentiated adenocarcinoma composed of large pleomorphic tumor cells with predominantly fine nuclear chromatin and prominent nucleoli (hematoxylin & eosin stain, 40×) with morphology identical to the patient's known lung adenocarcinoma—indicative of metastatic lesions (Figure 4). He was maintained on acid suppression. Unfortunately, his clinical status deteriorated, and he was eventually transitioned to comfort measures.

Lung cancer commonly metastasizes to the brain, liver, bones, and adrenal glands. Gastric metastases of lung cancer are rare and usually clinically silent. It may be diagnosed at the time of autopsy and is only infrequently captured endoscopically.^1^ Our case demonstrates that lung cancer can quickly progress, presenting with gastrointestinal bleeding and can appear as ulcerative nodules in an endoscopic evaluation.

## DISCLOSURES

Author contributions: All authors contributed to the conception, design, drafting, revision, and approval of the final article. AV Patel is the article guarantor.

Financial disclosure: None to report.

Informed consent was obtained for this case report.
